# Socioeconomic and regional differences in active transportation in Brazil

**DOI:** 10.1590/S1518-8787.2016050006126

**Published:** 2016-06-17

**Authors:** Thiago Hérick de Sá, Rafael Henrique Moraes Pereira, Ana Clara Duran, Carlos Augusto Monteiro

**Affiliations:** I Núcleo de Pesquisas Epidemiológicas em Nutrição e Saúde. Faculdade de Saúde Pública. Universidade de São Paulo. São Paulo, SP, Brasil; II Instituto de Pesquisa Econômica Aplicada. Brasília, DF, Brasil; III Transport Studies Unit. University of Oxford. Oxford, OX, UK; IV University of Illinois at Chicago. Chicago, IL, USA

**Keywords:** Motor Activity, Walking, Transportation, Urban Health, City Planning, Health Inequalities, Healthy City, Metropolitan Zones

## Abstract

**OBJECTIVE:**

To present national estimates regarding walking or cycling for commuting in Brazil and in 10 metropolitan regions.

**METHODS:**

By using data from the Health section of 2008’s *Pesquisa Nacional por Amostra de Domicílio* (Brazil’s National Household Sample Survey), we estimated how often employed people walk or cycle to work, disaggregating our results by sex, age range, education level, household monthly income *per capita*, urban or rural address, metropolitan regions, and macro-regions in Brazil. Furthermore, we estimated the distribution of this same frequency according to quintiles of household monthly income *per capita* in each metropolitan region of the country.

**RESULTS:**

A third of the employed men and women walk or cycle from home to work in Brazil. For both sexes, this share decreases as income and education levels rise, and it is higher among younger individuals, especially among those living in rural areas and in the Northeast region of the country. Depending on the metropolitan region, the practice of active transportation is two to five times more frequent among low-income individuals than among high-income individuals.

**CONCLUSIONS:**

Walking or cycling to work in Brazil is most frequent among low-income individuals and the ones living in less economically developed areas. Active transportation evaluation in Brazil provides important information for public health and urban mobility policy-making

## RESUMO

**OBJETIVO:**

Apresentar estimativas nacionais sobre o deslocamento a pé ou de bicicleta no trajeto casa-trabalho no Brasil e em 10 de suas regiões metropolitanas.

**MÉTODOS:**

Utilizando dados do Suplemento sobre Saúde da Pesquisa Nacional por Amostra de Domicílios de 2008, estimamos a frequência de pessoas empregadas que se deslocam a pé ou de bicicleta no trajeto casa-trabalho estratificada por sexo, e segundo faixa etária, escolaridade, renda domiciliar *per capita*, residência em área urbana ou rural, regiões metropolitanas e macrorregiões do país. Adicionalmente, estimamos a distribuição da mesma frequência segundo quintos da distribuição da renda domiciliar *per capita* em cada região metropolitana.

**RESULTADOS:**

Um terço dos homens e mulheres empregados desloca-se a pé ou de bicicleta de casa para o trabalho no Brasil. Em ambos os sexos, esta proporção diminui com o aumento da renda e da escolaridade e é maior entre os mais jovens, entre os que residem em área rural e naqueles residentes na região Nordeste. A depender da região metropolitana, a prática de deslocamento ativo entre os mais pobres é de duas a cinco vezes maior do que entre os mais ricos.

**CONCLUSÕES:**

O deslocamento a pé ou de bicicleta para o trabalho no Brasil é mais frequente entre os mais pobres e entre pessoas que vivem em áreas e regiões economicamente menos desenvolvidas. A avaliação do deslocamento ativo no País traz informações importantes para a discussão de políticas públicas de mobilidade.

## INTRODUCTION

Using active modes of transport in cities, such as walking or cycling, brings benefits to people’s health^10^ ^,^ ^30^ ^,^ ^31^ and to the population – examples include the reduction in pollution and traffic accidents^11^ ^,^ ^23^ ^,^ ^30^ ^,^ ^31^. Promoting active modes of transportation could also have a positive economic impact, besides the direct impact on health^13^.

The promotion of active transport is greatly favored by the understanding of the frequency and distribution of this practice in specific contexts. The most common active transport modes, such as walking or cycling, are related to individual factors (age, sex, income, education), environmental factors (climate, topography, and built environment), and characteristics that are specific to each itinerary, such as the distance to be covered, the reason for the travel, and its cost^2^ ^,^ ^22^. Taken together, these factors help us understand why the proportion of people who use active transport varies across countries^1^ ^,^ ^9^, regions or cities^12^ ^,^ ^25^, and socioeconomic strata of the population^14^. Studies suggest that city infrastructures also determine the habits of walking or cycling^28^, besides promoting higher safety^23^. Nonetheless, the extent of these effects varies according to income and age^14^.

There is a lack of national estimates on the frequency and distribution of active transport in Brazil. A great deal of the country’s literature is limited to local studies, mainly in the South and Southeast regions^29^. Disclosing these data may contribute to the planning of policies and programs that consider regional characteristics of active transportation, as well as national strategies to foster these practices across the socioeconomic spectrum. This study aimed to provide national estimates on the frequency and distribution of walking or cycling for commuting in Brazil.

## METHODS

### Data Source

This study used data from the Health section of *Pesquisa Nacional por Amostra de Domicílio* (PNAD – Brazil’s National Household Sample Survey), which was conducted by the Brazilian Institute of Geography and Statistics (IBGE) in 2008^a^. PNAD is the only annual sample survey with public nationwide data on commuting to work^a^.

Its section on health from 2008 investigated topics related to the population’s health, among which the practice of physical activity in different domains (physical leisure activities, commuting to work, professional activities, and heavy cleaning in the household environment).

### Sample plan and data collection

The PNAD sample from 2008 was obtained through complex probability sampling by clusters in two or three stages: municipality, census tract, and household. In the first and second stages, the spatial units (municipality and census tracts, respectively) were selected with replacement and with probability proportional to the population in 2000’s Brazilian Population Census^a^. In the third stage, households were randomly selected according to the number of households in each census tract. All the household residents were interviewed or had their information disclosed by other members of their families. Employing appropriate weighting factors, the data from PNAD 2008 allow estimates that are representative of the total Brazilian population, urban or rural populations, and of the populations in macro-regions, states, and metropolitan regions in Brazil.

The questionnaire from PNAD 2008 included questions regarding commuting to work by bicycle or on foot and the time spent on commuting. Individuals 13 year old or younger or those with health problems that prevented them from walking approximately 100 meters or shopping for groceries, clothes, or medications without help, were excluded from the survey.

### Study variables and data analysis

An indicator was created for the practice of active transport to work by calculating the share of employed people aged 14 years or older who walk or cycle to work, regardless of the trip duration. This indicator and its corresponding 95% confidence interval (95%CI) were separately estimated for men and women considering differences by sex previously observed in the Brazilian population^7^ ^,^ ^15^.

These estimates were calculated by sex considering their age (15-24; 25-34; 35-44; 45-54; 55-64, and ≥ 65 years), education level (1-3; 4-7; 8-10; 11-14, and ≥ 15 years of study), macro-region of residence (North; Northeast; South; Southeast, and Midwest), whether they lived in a macro-region (yes; no), metropolitan region (Belem; Belo Horizonte; Curitiba; Distrito Federal; Fortaleza; Porto Alegre; Recife; Rio de Janeiro; Salvador; and Sao Paulo), urbanicity (rural or urban areas), and household monthly income *per capita* (in decile groups).

Furthermore, for participants living in the nine largest metropolitan regions in Brazil or the Federal District, we estimated the distribution of walking or cycling to work according to the quintiles of household income *per capita*, calculated for each urban area. The frequency and distribution of people whose active commute lasts 30 minutes or longer – a commonly used indicator in the literature^3^ ^,^ ^7^ ^,^ ^15^ ^,^ ^18^ ^,^ ^27^ – are available upon request and on personal storage websites such as Research Gate.

The differences observed between groups were considered to be statistically significant when the specific estimate of one of them was not contained in the interval estimate of the other, namely, 95%CI^16^. The study followed the principles of the Declaration of Helsinki, and was approved by the Research Ethics Committee of the Faculdade de Saúde Pública of the Universidade de São Paulo. The analytical procedures in this study were executed on R statistical package (2.15.3). We used by the survey package in order to consider PNAD 2008’s design and sample weights.

## RESULTS

The Table shows estimates for active transport for men and women by urbanicity, metropolitan region, and macro-region. Around one third of Brazil’s male and female populations actively commute. In the country’s metropolitan regions, this share drops to a little less than 20.0%. The highest shares of active travel were found among those residing in rural areas, whereas the smaller shares were observed in the Northeast and Southeast macro-regions. In general, the share of men and women who use active transport modes to work is similar in all analyzed subgroups, with the exception of those who live in rural areas and in the North macro-region. There, more men and women walk or cycle to work.

**Table:** Share of the population of 14 years of age or older who walk or cycle to work, according to sociodemographic variables. Brazil, 2008.

Sociodemographic characteristic	Men			Women		
	
	n (thousands)	%	95%CI	n (thousands)	%	95%CI
Household status						
Rural*	5,271	53,4	50,8–55,9	2,666	45,2	42,7–47,9
Urban*	12,055	29,5	29,0–30,1	9,692	31,0	30,4–31,7
Metropolitan region						
No	14,556	40,4	39,4–41,3	10,178	40,0	39,1–40,9
Yes	2,769	18,9	18,3–19,6	2,181	18,7	18,0–19,4
Macro-region						
Midwest*	774	24,2	22,7–25,7	615	26,5	25,0–28,1
Northeast*	6,365	46,9	45,2–48,5	3,859	40,5	39,0–42,0
North*	1,644	39,5	35,9–43,2	922	35,8	33,4–38,3
Southeast*	5,669	26,7	26,0–27,5	4,505	28,1	27,1–29,2
South*	2,648	33,8	32,0–35,6	2,291	37,2	35,5–38,9
Total	17,325,1	34,2	33,5–34,9	12,358,5	33,3	32,6–34,0

**Figure 1:**
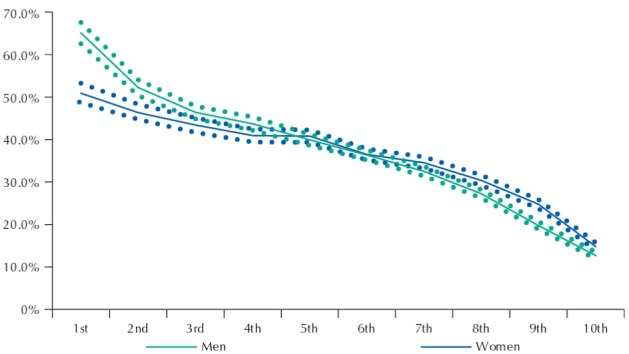
Frequency (%) of people walking or cycling to work among men and women and according to income deciles*. Brazil, 2008.

Source: PNAD (IBGE).

**Figure 2:**
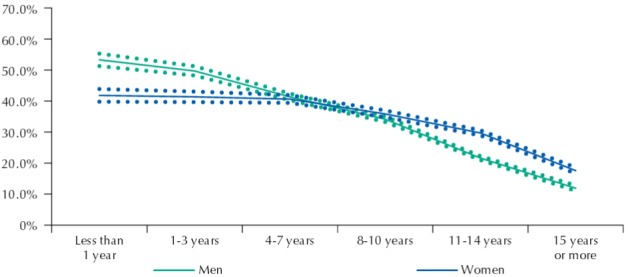
Frequency (%) of people walking or cycling to work among men and women and according to years of study. Brazil, 2008.

Source: PNAD (IBGE).

**Figure 3:**
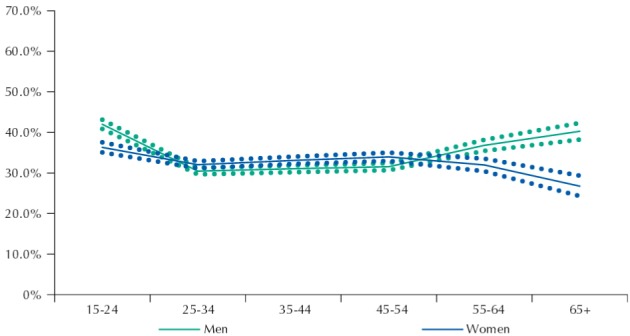
Frequency (%) of people walking or cycling to work among men and women and according to age ranges. Brazil, 2008.

Source: PNAD (IBGE).

* Significant differences between men and women,

Figures 1 to 3 show the frequency of active transport to work among men and women by income deciles, education, and age, respectively.

* Household monthly income *per capita* deciles.

The share of people who walk or cycle to work decreases as income and education levels rise for men and women (Figures 1 and 2). However, such reduction is more prominent among men, contributing with our findings that point to more highly educated and high-income women than men actively commuting to work.

A higher frequency of active travel to work was observed among teenagers (14 to 19 years of age). Significant differences by sex were only observed among adults who were 55 years or older (Figure 3).

**Figure 4:**
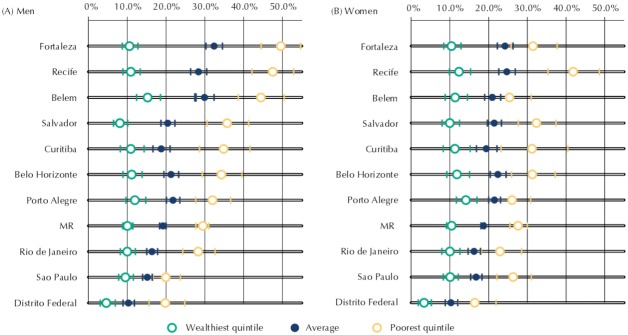
Share of employed men (A) and women (B) who walk or cycle to work, according to income quintiles. Brazilian metropolitan regions, 2008.

Source: PNAD (IBGE).

Figure 4 shows the share of men and women who walk or cycle to work by quintiles of household monthly income *per capita* in the 10 metropolitan regions. In all urban areas, the quintile of the population with the lowest income was found to have higher active transport levels (between two and five times higher) than the wealthiest portion of the study population. Nonetheless, the differences between groups at the extremes of the income distribution varied considerably between men and women and across metropolitan regions. The largest differences were observed in the male population residing in metropolitan regions located in Brazil’s North and Northeast regions.

MR: Metropolitan regions

* Household monthly income *per capita* deciles.

The smallest frequency of men and women who walk or cycle to work was found in the Federal District, for both the wealthiest (Q5) and the poorest portions of the sample (Q1). The Federal District had the smallest difference by income level in the country (Figure 4).

## DISCUSSION

Socioeconomic and regional differences were found in active transport to work in Brazil, and it was observed to be the most common practice among low-income individuals and in the least economically developed areas (rural area; non-metropolitan regions; North and Northeast macro-regions; and metropolitan regions of Belem, Recife, and Fortaleza). The inverse association of income and active transport was also found in other Brazilian^6^ ^,^ ^7^ ^,^ ^25^ ^,^ ^27^ ^,^ ^b^ and international studies^2^.

The results of this study show one rare example of health inequalities that favor lower-income individuals. The inverse relationship between the practice of active travel and people’s income levels possibly reflects budget constraints of low-income families, the gentrification in Brazilian cities, and poor public transportation conditions. Not necessarily a practice that is motivated by socioenvironmental and health benefits^6^ ^,^ ^26^ ^,^ ^c^ ^,^ ^d^. Spatial segregation, coupled with an inefficient public transportation system, contributes to increase longer distances covered on foot or by bicycle as it increases the time it takes people to access the public transportation system and its connections^d^.

When we compare Brazil’s 10 metropolitan regions, we identify little variation in the frequency of active transport among the 20.0% wealthiest people in the population (with the exception of the Federal District). On the other hand, among individuals in the first quintile of household income, a higher prevalence of active commuting was found in the least economically developed metropolitan regions, where transportation systems deteriorated worse over the last decades, such as Belem, Fortaleza, and Recife^21^. Considering the maintenance of the current spatial structure of cities and transportation systems, along with rising income and purchasing power conditions of low-income individuals in Brazil^19^, levels of walking or cycling to work are expected to decrease and get closer to the homogeneously low level observed among high-income individuals. Comparing Brazil’s Federal District to other metropolitan regions, the smaller share of active transport both among the wealthiest and the poorest individuals reflects Brasilia’s unique urban planning – which is largely car-oriented - and the long distances a great deal of the population need to cover between the satellite cities and Brazilian capital, where most of the jobs are located^17^.

Another reason that leads us to believe in the worsening of the indicator is the expansion of household car ownership^e^. The rising income in Brazil from 2004-2014 was accompanied by an increase that is more than proportional in transportation expenditures, especially among low-income individuals^e^. Furthermore, despite this substantial growth in motorization rates, which took place in North and Northeast large cities, these rates still correspond to half of those observed for other Brazilian metropolitan regions^21^. Although time series with nationally representative information on active transport in Brazil are unavailable, a documented reduction in Brazilian major cities has been reported^18^.

A similar situation can be observed in rural areas, especially due to the increasing sales of motorcycles^17^ ^,^ ^19^. Despite the lack of detailed information on the evolution of motorized trips in rural areas, the highest fleet growth between 2001 and 2012 was observed in municipalities of up to 20,000 inhabitants (an approximate 400.0% increase), which was higher that the growth rate observed for automobiles (143.0%)^c^. However, mobility policies that focus on improving and integrating several modes of transport may help increase the rates of active commute already shown in other contexts^3^ ^,^ ^5^ ^,^ ^8^.

Approximately a third of men and women actively commute to work in Brazil, similar to European countries such as France (34.9%) and Holland (37.9%), and below the rates found in China (46.1%)^9^. Active transportation was more prevalent among men only in a few metropolitan regions (Recife, Belem, and Fortaleza), in rural areas, and among the oldest (55 years of age or older); and it is more frequent among women in the highest income and education level strata. Issues regarding the adoption of healthier habits and differences concerning the ownership of vehicles inside one’s household may be driving gender-related differences^4^. Such differences may also contribute to explain the slower increase in obesity rates among women of higher education levels as compared to equally educated men^20^. More detailed studies on the relationships between household chores, out-of-home activities, and gender roles in the determination of the practice of walking or cycling to and from work in Brazil are required to confirm these hypotheses.

This study has some limitations. They include the impossibility of including walking or cycling for other reasons other than work. Nevertheless, commuting for work accounts for approximately 45.0% of all trips in Brazilian metropolitan regions^f^. Additionally, PNAD’s data do not allow us to evaluate walking and cycling separately or to explore covered distances, nor to which extent these practices vary within the same city. These issues would be extremely relevant for impact evaluation of public policies that seek to increase the share of non-motorized transportation modes.

This is, however, one of the first studies that used PNAD’s new weighting factors, which allowed us to estimate walking or cycling to work for the entire country, besides describing differences in such practice by socioeconomic and geographic variables.

Recent socioeconomic changes that took place in Brazil influenced the population’s access to automobiles and motorcycles. However, the access to public services (for example, public transportation) did not occur at the same rate. Our study contributes to the understanding of health inequalities in Brazil and brings elements to urban mobility policy-making that focus on the integration of transport modes and on increasing the share of non-motorized trips considering socioeconomic and regional differences. The practice of active commuting has marked regional and socioeconomic differences in Brazil. Current national and regional policies, such as Brazil’s National Urban Mobility Policy^g^ and city-level Master plans should consider our findings in shaping active commuting levels across the country. Finally, policy makers must ensure that low-income individuals are not affected by a necessary migration to motorized transport modes by reducing distances of daily commute as well as improving their access to adequate dedicated infrastructure for walking and bicycling, and to public transportation.

## Footnotes

a Instituto Brasileiro de Geografia e Estatística. Pesquisa Nacional por Amostra de Domicílios 2008: um panorama da saúde no Brasil: acesso e utilização dos serviços, condições de saúde e fatores de risco e proteção à saúde. Rio de Janeiro; 2010 [cited 2016 Feb 26]. Available from: http://www.ibge.gov.br/home/estatistica/populacao/panorama_saude_brasil_2003_2008/default.shtm

b Companhia do Metropolitano de São Paulo. Pesquisa Origem e Destino 2007: Região Metropolitana de São Paulo: síntese das informações da pesquisa domiciliar*.* São Paulo: Diretoria de Planejamento e Expansão dos Transportes Metropolitanos; 2008 [cited 2016 Feb 26]. Available from: http://www. OD2007/sintese_od2007.pdf metro.sp.gov.br/metro/arquivos/

c Institutos Nacionais de Ciência e Tecnologia, Observatório das Metrópoles. Evolução da frota de automóveis e motos no Brasil 2001 – 2012 (relatório 20213). Rio de Janeiro; 2013 [cited 2016 Feb 26]. Available from: http://www.observatoriodasmetropoles.net/download/auto_motos2013.pdf

d ONU Hábitat –Programa de las Naciones Unidas para los Asentamientos Humanos. Estado de las ciudades de América Latina y el Caribe 2012: rumbo a una nueva transición urbana. Kenia; 2012 [cited 2016 Feb 26]. Available from: http://www.onuhabitat.org/index.php?option=com_docman&task=doc_ view&gid=816&tmpl=component&format=raw&Itemid=538

e Associação Brasileira dos Fabricantes de Motocicletas, Ciclomotores, Motonetas, Bicicletas e Similares. Anuário da Indústria Brasileira de Motociclos. São Paulo (SP): ABRACICLO; 2011.

f Corporación Andina de Fomento. Desarrollo urbano y movilidad en América Latina. Ciudad de Panamá: CAF - Observatorio de la Movilidad Urbana; 2011 [cited 2016 Feb 27]. Available from: http://www.caf.com/media/4203/desarrollourbano_y_movilidad_americalatina.pdf

g Brasil. Lei n 12.587, de 3 de janeiro de 2012. Institui as diretrizes da Política Nacional de Mobilidade Urbana; revoga dispositivos dos Decretos-Leis nºs 3.326, de 3 de junho de 1941, e 5.405, de 13 de abril de 1943, da Consolidação das Leis do Trabalho (CLT), aprovada pelo Decreto-Lei nº 5.452, de 1º de maio de 1943, e das Leis nºs 5.917, de 10 de setembro de 1973, e 6.261, de 14 de novembro de 1975; e dá outras providências. *Diario Oficial Uniao*. 4 jan 2012; Seção 1: 1.
